# Endovascular Intervention for Acute Ischemic Stroke in Light of Recent Trials

**DOI:** 10.1155/2014/429549

**Published:** 2014-11-03

**Authors:** Kenan Alkhalili, Nohra Chalouhi, Stavropoula Tjoumakaris, David Hasan, Robert M. Starke, Mario Zanaty, Robert H. Rosenwasser, Pascal Jabbour

**Affiliations:** ^1^Department of Surgery, University of Arizona, Tucson, AZ Department of Surgery, University of Arizona, Tucson, AZ 85721, USA; ^2^Department of Neurosurgery, Thomas Jefferson University and Jefferson Hospital for Neuroscience, Philadelphia, PA 19107, USA; ^3^Department of Neurosurgery, University of Iowa, Iowa City, IA 52242, USA; ^4^Department of Neurosurgery, University of Virginia, Charlottesville, VA 22908, USA; ^5^Department of Neurological Surgery, Division of Neurovascular Surgery and Endovascular Neurosurgery, Thomas Jefferson University Hospital, 901 Walnut Street, 3rd Floor, Philadelphia, PA 19107, USA

## Abstract

Three recently published trials, MR RESCUE, IMS III, and SYNTHESIS Expansion, evaluating the efficacy and safety of endovascular treatment of acute ischemic stroke have generated concerns about the future of endovascular approach. However, the tremendous evolution that imaging and endovascular treatment modalities have undergone over the past several years has raised doubts about the validity of these trials. In this paper, we review the role of endovascular treatment strategies in acute ischemic stroke and discuss the limitations and shortcomings that prevent generalization of the findings of recent trials. We also provide our experience in endovascular treatment of acute ischemic stroke.

## 1. Introduction

About 795 000 people experience a new or recurrent stroke in the United States on annual basis [1]. Ischemic strokes represent 85% of cases and are frequently caused by occlusion of larger arteries [1–3]. The role of endovascular therapy in the treatment of acute ischemic stroke (AIS) must be carefully considered, given the remarkable advancement that imaging and endovascular treatment modalities have undergone over the past several years. Recent publication of the Interventional Management of Stroke (IMS) III [4], Mechanical Retrieval and Recanalization of Stroke Clots Using Embolectomy (MR RESCUE) [5], and SYNTHESIS Expansion [6] trials brought into question the clinical value of endovascular therapy in acute ischemic stroke (AIS). These reports, however, have several significant weaknesses and do not reflect the recent advances in imaging and endovascular technology.

The endovascular treatment of AIS has advanced significantly, with notable transition from intra-arterial (IA) chemical thrombolysis to mechanical thrombectomy [7–10]. First-generation devices, such as the Merci (Stryker; Kalamazoo, MI, USA) and Penumbra (Penumbra Inc., Alameda, CA, USA) devices, were developed to engage or aspirate the clot in different means [11]. Aspiration through the balloon guide catheter is applied during clot retrieval. The second-generation devices (e.g., “stentrievers” such as Solitaire (Covidien/ev3, Dublin, Ireland) or Trevo (Stryker, Kalamazoo, MI, USA)) have unique dual functionality, acting as a temporary bypass providing immediate flow restoration through the thrombus and as a clot retriever, trapping thrombus into its cells [12, 13].

The effect of stent retrievers on acute stroke treatment is currently being investigated in several ongoing multicenter trials such as the STAR Trial (Solitaire FR Thrombectomy for Acute Revascularization), the THRACE trial, and the RIVER II trial.

## 2. IMS III

### 2.1. Design

IMS III trial is an international, phase 3, randomized, open-label clinical trial with a blinded outcome that tested the approach of intravenous recombinant tissue plasminogen activator (rtPA) followed by protocol-approved endovascular treatment, as compared with standard intravenous rtPA.

The trial hypothesized that combined IV-IA approach (“bridging”) for recanalization of acute stroke is superior to standard IV approach.

### 2.2. Results

Functional independence scores did not differ between the endovascular group and IV rtPA cohorts (modified Rankin scores [mRS] of 0–2: 40.8% versus 38.7%) as did mortality rates (19.1% and 21.6%, *P* = 0.52) at 3-month follow-up. Rates of symptomatic intracranial hemorrhage (ICH) were similar between both groups refuting safety concerns about endovascular therapy. On the other hand, in a subgroup analysis in patients with confirmed large vessel occlusion (LVO), combined therapy was superior in terms of recanalization and outcomes to IV rtPA alone (*P* = 0.01).

### 2.3. Flaws

A number of weaknesses have been observed in this trial [14–16]. To begin with, during the 6-year long inclusion period, imaging and endovascular treatment modalities underwent major advancement, and utilization of new technologies was only partially adopted. Only 47% of the patients had CT angiogram (CTA) imaging at the time of enrollment, since the National Institute of Health Stroke Scale (NIHSS) score alone is not an accurate enough predictor of LVO. Subsequently, patients without confirmed LVO have been enrolled in the trial. Twenty percent of patients enrolled in the endovascular arm had no LVO on angiography; these patients were still considered in IA arm for trial purposes.

Another limitation of IMS trial is that most patients in the endovascular arm received IA thrombolysis or thrombectomy using Merci retriever. Both approaches are currently considered outdated for endovascular treatment of AIS. The first-generation thrombectomy devices used in the trial are known to be inferior to stent retrievers in terms of recanalization and outcome [17, 18]. Thus, the results of IMS III did not reflect the huge advances that occurred in endovascular technology over the past few years.

In addition, the significant delay of more than 2 hours between initiation of IV and IA merits attention. This delay may have a deleterious effect on the IA intervention efficacy. Moreover, most of the patients enrolled in IA arm received less than the standard dose of IV rtPA before undergoing angiography, and although all patients in the IV arm received standard doses, we should also mention the selection bias that limited IA treatment to only IV Tpa eligible patients, where in real life the majority of patients eligible for IA thrombectomy did not meet criteria for IV tPA.

### 2.4. Strengths

This trial is considered to be the largest randomized stroke trial conducted so far. Nine hundred subjects were planned to demonstrate the superiority of combined approach. After the enrolment of 656 participants, the study was halted because interim analysis showed futility (low probability of finding significant difference between both groups). However, when only patients with confirmed LVO were considered, there was a significant benefit for endovascular therapy in terms of recanalization and outcomes. This is probably the single most important conclusion of the IMS III trial.  Table 1 summarizes recently published randomized trials of acute stroke therapies.

## 3. MR RESCUE

### 3.1. Design

MR RESCUE was a phase 2b, randomized, controlled, open-label (blinded outcome), multicenter trial. The trial hypothesized that patients with favorable neuroimaging “penumbral” pattern were more likely to achieve better outcome from IA treatment of AIS. Penumbral pattern was defined as the presence of substantial salvageable tissue with small infarct core (predicted infarct core ≤90 mL and ratio of predicted infarct tissue within the at-risk region ≤70%). Nonpenumbral pattern was defined as the presence of larger core or small or absent penumbra. The study randomized patients into 4 groups based on perfusion results (penumbral versus nonpenumbral) and type of treatment (medical versus endovascular). Patients with confirmed LVO were randomly assigned within 8 hours after the onset of symptoms to undergo either mechanical embolectomy (Merci retriever or Penumbra system) or standard medical care.

### 3.2. Results

Embolectomy and standard medical care exhibited similar rates of mRS (3.9 versus 3.9, *P* = 0.99). Moreover, there was no significant difference between embolectomy and standard medical care in patients with penumbral pattern versus nonpenumbral pattern. On the 3-month mRS, there was no interaction between the pretreatment imaging pattern and treatment assignment (*P* = 0.14). However, the final infarct volume was lower in patients with a favorable penumbral pattern regardless of treatment assignment.

### 3.3. Flaws

A number of limitations that question the trial conclusions should be considered [14–16]. First and foremost, MR RESCUE only included first-generation endovascular thrombectomy technologies, and higher efficacy retrievers were not utilized. Secondly, the small number of patients assigned to each of the 4 groups has likely underpowered this trial. Importantly, effective recanalization was not achieved in most endovascular group patients, regardless of penumbral imaging pattern. Only 16 of 64 patients (27%) achieved TICI 2b or 3 reperfusion. This rate is obviously not in line with acceptable treatment standards for acute ischemic stroke. The goal of any endovascular stroke intervention should be to achieve arterial recanalization. It is therefore impossible for MR RESCUE to comment on the efficacy of endovascular stroke therapy when only a minority of patients achieved arterial recanalization.

### 3.4. Strengths

**Figure 1:**
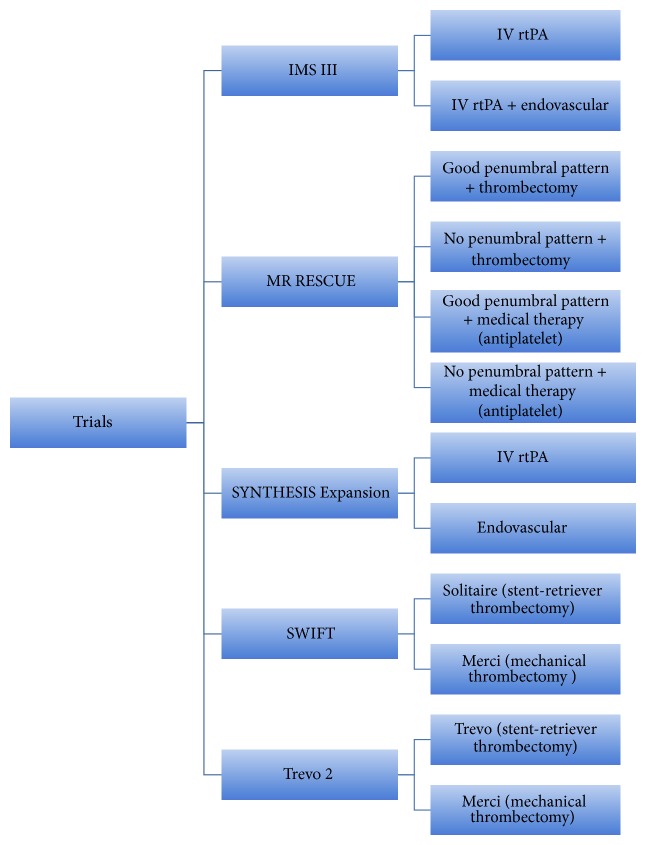
Trial arms of recently published randomized trials of acute stroke treatment.

Unlike the IMS III or SYNTHESIS trials, pretreatment evaluation was more precise. CTA or MRA was used to depict LVO, and multimodal CT or MR imaging of the brain was used to evaluate penumbral status (Table 1, Figure 1).

## 4. SYNTHESIS Expansion

### 4.1. Design

It is a multicenter Italian trial that randomly assigned 362 patients presenting with an AIS. The trial compared the treatment with IV rtPA within 4.5 hours of onset versus IA therapy within 6 hours of onset.

### 4.2. Results

There was no significant difference between both trial arms in terms of safety (rates of intracranial hemorrhage and death) or long-term outcomes.

### 4.3. Flaws

The trial had a number of weaknesses that question the validity of its results [14–16]. CTA or MRA was not obtained to confirm LVO or evaluate penumbra. Additionally, there was no lower threshold of NIHSS defined for study inclusion present. Around half of the patients enrolled had an NIHSS of 10 or less. Patients with NIHSS score as low as 2 were included; such patients are more likely to have a good recovery at 3 months regardless of the treatment given [16]. About 10% of patients assigned to intervention did not exhibit an LVO at angiography. Still, those patients were exposed to the potential risks of an intervention with injection of thrombolytics in the suspected target vessel.

Most of the patients in endovascular arm were treated with either wire manipulation or local thrombolysis. Only 10% received stent retrievers as a treatment. Additionally, the endovascular group received treatment 1 hour later than the IV tPA group, which might explain the comparable results of endovascular treatment and IV tPA treatment. Given this, it is expected that the SYNTHESIS study reconfirms the IMS III outcomes regarding the limited efficacy of endovascular treatment performed with outdated methods (Table 1, Figure 1).

## 5. Recent Trials Showing Superiority of Stent Retrievers

### 5.1. SWIFT

SWIFT was a multicenter, randomized, prospective, parallel-group trial with blinded primary endpoint ascertainment [17]. SWIFT was designed to provide definitive information on the efficacy and safety of the Solitaire Flow Restoration device in comparison to first-generation Merci retriever. It was the first trial to conduct a direct, randomized comparison of one mechanical thrombectomy device to another.

Patients were eligible if they had AIS with moderate to severe neurological deficits, harbored angiographically confirmed occlusions of proximal cerebral arteries. Inclusion criteria were NIHSS score (≥8 and ≤30) and ineligibility for or failure to respond to intravenous rtPA.

The primary efficacy outcome was achieved more often in the Solitaire group than it was in the Merci group (61% versus 24). More patients had better 3-month neurological outcome with Solitaire than with Merci (58% versus 33%); 90-day mortality was lower in the Solitaire group than it was in the Merci group (17 versus 38). Thus, the Solitaire Flow Restoration Device was associated with substantially better angiographic, safety, and clinical outcomes compared to the Merci Retrieval System (Table 1, Figure 1).

### 5.2. Trevo 2

Trevo 2 was a randomized, prospective, controlled, multicenter, open-label trial [18]. The trial aimed to compare the efficacy and safety of mechanical thrombectomy using Trevo retriever with that of the Merci retriever in AIS. Patients were recruited from 26 sites in the USA and one in Spain. Participants had angiographically confirmed LVO strokes and NIHSS scores of 8–29 within 8 h of symptom onset. Eighty-six percent of patients in the Trevo group and 60% in the Merci group have shown successful recanalization, defined as TICI 2 or greater flow in the territory of the occlusion. The Trevo retrievers showed superior neurologic outcome compared to Merci device; however, the mortality rates were comparable.

The results of these 2 trials highlight again the superiority of stent retrievers to older devices in acute stroke intervention. Thus, any trial assessing the safety and efficacy of endovascular therapy should have stent retrievers as the main treatment modality (Table 1, Figure 1).

### 5.3. Our Experience

Two different studies on endovascular intervention for different age groups were conducted at the senior author institution [19, 20].

#### 5.3.1. Young Patients with Large Vessel Occlusions

Young patients (<55 years old) undergoing endovascular intervention for acute ischemic stroke were included in this study. Patients included had confirmed LVO, NIHSS greater than or equal to 5–8, and evidence of a large penumbra on CT perfusion. The mean admission NIHSS score was 14.1 (median 13.5). Intravenous rtPA was administered before initiation of endovascular therapy in half of the patients.

About one-third of the patients received second-generation retrievers, and the other two-thirds were treated with first-generation devices. Successful recanalization was achieved in as many as 93% of patients, and more than 90% achieved ≥II TIMI. Favorable outcome mRS (0–2) was achieved in up to 73% of patients. Interestingly, second-generation retrievers (Solitaire) showed higher rates of favorable outcome compared to the first-generation devices. More importantly, endovascular treatment tended to be even more effective in younger patients <35 compared to those >35. These findings suggest that endovascular treatment for AIS has a particularly favorable outcome in young patients and support aggressive interventional strategies in this age group.

#### 5.3.2. Septuagenarians and Above

A retrospective chart review was conducted to include patients over 75 years old treated for AIS. Inclusion criteria for intervention were a minimum NIHSS of 8, CT perfusion with ischemic penumbra on mean transient time and cerebral blood flow but preservation of cerebral blood volume, and detectable arterial occlusion on CT angiography.

Eight (16%) patients underwent stent placement after intra-arterial thrombolysis, 10 (20%) underwent balloon angioplasty, and seven (14%) underwent both angioplasty and stent placement. Twenty-one (41%) required only intra-arterial thrombolytics. An improvement in thrombolysis score was noted in 67% of the patients. The average mRS score on discharge was 3.9. Symptomatic intracranial hemorrhage occurred in 6% of the patients. Two fatalities resulted from intraoperative vessel rupture (3.9%).

Due to strict inclusion criteria for intervention given the increased risks of endovascular treatment in this age group, the patient population is rather small. Moreover, patient outcome at discharge may also not reflect ultimate outcome. Another limitation to our study is the lack of shift analysis of mRS, that is, assessment of mRS improvement rather than recording the good outcome only. Larger prospective randomized studies are required to investigate acute stroke interventions in this patient population.

Authors concluded that multimodal endovascular recanalization of AIS is a relatively safe treatment option in patients older than 75 years of age. However, careful patient selection by clinical and radiographic inclusion criteria is necessary for the successful management of stroke in this age group.

#### 5.3.3. Evaluation of the Triage Protocol for Acute Ischemic Stroke

We conducted a retrospective review on 132 patients, 94 of which were undergoing CTP-guided and 38 undergoing time-guided (maximum 8 h from symptom onset) mechanical recanalization [21]. The study aimed at comparing the safety and efficacy of CTP-guided to time-guided mechanical recanalization in AIS. We observed no difference in the partial-to-complete recanalization rate between the CTP and the non-CTP group. However, the non-CTP group experienced a significantly higher rate of ICH. Multivariate analysis revealed CTP-guided patient selection to be an independent negative predictor of in-hospital mortality (OR = 3.2, *P* = 0.01), suggesting a potential benefit to using CTP for triage. Perfusion studies have now become the preferred triage method at our institution. We believe that, for stroke interventions to be effective, advanced imaging techniques such a CTP should be used to identify patients who can benefit from treatment.

## 6. Conclusions

IMS III, MR RESCUE, and SYNTHESIS Expansion trial results in the New England Journal of Medicine raised the doubts about the clinical value of intra-arterial (IA) therapy for the treatment of AIS. However, each has significant limitations that prevent its generalization to contemporary AIS treatment. Failure to adequately identify LVOs in IMS III and SYNTHESIS trials is a major drawback. Both IMS III * *and SYNTHESIS trials failed to use appropriate imaging technique (e.g., CTA, MRA) to confirm LVO. Moreover, salvageable brain evaluation with CT perfusion or MR was not conducted.

The second major drawback is the predominant use of the first-generation thrombectomy devices in the three trials. Subsequently, the revascularization rate did not meet the standards, particularly in MR RESCUE trial where it was dramatically worse than recent SWIFT and Trevo trials using second-generation devices.

Moreover, delays in treatment further biased the trials in IA therapy; treatment was initiated approximately 1-2 hours later in the IA arm compared with the IV arm.

Analysis of the IMS III, SYNTHESIS, and MR RESCUE studies was informative in terms of improvements that should be implemented in future studies. The future evaluation of endovascular therapy in AIS has to consider proper selection of patients (confirmed large vessel occlusion, evaluation of salvageable brain) and the homogenous use of up-to-date treatment methods (e.g., second-generation retrievers). It is important to reiterate that when only patients with confirmed LVOs were included in the analysis of the IMS III, endovascular therapy showed a significant clinical benefit.

Our experience at our institution has confirmed remarkably high rates of arterial recanalization and favorable outcomes in young patients, presenting with AIS and LVO. Thus, aggressive intervention strategy in these patients is recommended. Furthermore, in patients older than 75 years, multimodal endovascular recanalization of AIS has proved to be a relatively safe treatment option. However, careful patient selection by clinical and radiographic inclusion criteria is necessary for the successful management of stroke in this age group.

## Figures and Tables

**Table 1:** Recently published randomized trials of acute stroke therapies (design, limitations, and conclusions).

Study	Subjects	Centers	Trial arms	Types of treatment	Flaws	Conclusions
IMS III	656	58	(1) IV rtPA; (2) IV rtPA + endovascular	tPA (0.9 mg per kilogram) Merci retriever, Penumbra system, and Solitaire FR stent retriever	(1) Only 47% of enrolled patients had CT angiogram imaging at the time of enrollment (2) 20% of patients assigned to the IA treatment arm had no LVO or had thrombus that was inaccessible by catheter therapies (3) Significant majority in IA arm received less than the standard dose of IV rtPA before undergoing angiography (4) Utilization of first-generation thrombectomy devices	Comparable safety outcomes and no significant difference in functional independence with endovascular therapy after intravenous tPA, as compared with intravenous tPA alone

MR RESCUE	127	22	(1) Good penumbral pattern + thrombectomy (2) No penumbral pattern + thrombectomy (3) Good penumbral pattern + medical therapy (4) No penumbral pattern + medical therapy	Merci retriever, Penumbra system	(1) Only included first-generation endovascular thrombectomy technologies (2) The small number of patients assigned to each of the 4 groups has likely underpowered this trial (3) Only 16 of 64 patients (27%) achieved TICI 2b or 3 reperfusion	(1) A favorable penumbral pattern on neuroimaging did not identify patients who would differentially benefit from endovascular therapy (2) Embolectomy has not shown to be superior to standard care, in terms of safety and functional outcomes

SYNTHESIS Expansion	362		(1) IV rtPA (2) Endovascular	Wire manipulation, IA rtPA, Merci retriever, Penumbra system, and Solitaire stent retrievers	(1) No preprocedural imaging was obtained to confirm LVO (2) No defined lower threshold of NIHSS; 10% of patients in the SYNTHESIS trial randomly assigned to intervention did not harbor an LVO at angiography (3) Treatment was initiated approximately 1 hour later in the IA arm compared with the IV arm (4) Failing to report the revascularization (TICI)	No difference in functional outcome, safety, or mortality between the trial arms

SWIFT	144	18	(1) Solitaire (stent-retriever thrombectomy) (2) Merci (mechanical thrombectomy)	Solitaire stent retriever, Merci retriever	—	The Solitaire Flow Restoration Device achieved substantially better angiographic, safety, and clinical outcomes than did the Merci Retrieval System

Trevo 2	178	27	(1) Trevo (stent-retriever thrombectomy) (2) Merci (mechanical thrombectomy)	Trevo stent retriever, Merci retriever		Trevo retrievers achieved substantially better angiographic and clinical outcomes than did the Merci Retrieval System; safety endpoint did not differ between the trial arms
